# Phase I study of ipatasertib as a single agent and in combination with abiraterone plus prednisolone in Japanese patients with advanced solid tumors

**DOI:** 10.1007/s00280-019-03882-7

**Published:** 2019-06-21

**Authors:** Toshihiko Doi, Yutaka Fujiwara, Nobuaki Matsubara, Junichi Tomomatsu, Satoru Iwasa, Akari Tanaka, Chihiro Endo-Tsukude, Shintaro Nakagawa, Shunji Takahashi

**Affiliations:** 1 Department of Experimental Therapeutics, National Cancer Center Hospital East, 6-5-1 Kashiwanoha, Kashiwa-shi, Chiba-ken 277-8577 Japan; 2 Department of Experimental Therapeutics, National Cancer Center Hospital, Tokyo, Japan; 3 Department of Breast and Medical Oncology, National Cancer Center Hospital East, Kashiwa, Japan; 4 Department of Medical Oncology, The Cancer Institute Hospital of Japanese Foundation for Cancer Research, Tokyo, Japan; 5 Clinical Science and Strategy Department, Chugai Pharmaceutical Co., Ltd, Tokyo, Japan; 6 Clinical Pharmacology Department, Chugai Pharmaceutical Co., Ltd, Tokyo, Japan; 7 Clinical Information and Intelligence Department, Chugai Pharmaceutical Co., Ltd., Tokyo, Japan

**Keywords:** Dose-limiting toxicity, Ipatasertib, Pharmacokinetics, Prostate cancer, Akt inhibitor, PTEN

## Abstract

**Purpose:**

Ipatasertib is a selective inhibitor of Akt, a frequently activated protein kinase in human cancers. The current study assessed the safety, tolerability, and pharmacokinetics of ipatasertib in Japanese patients with solid tumors.

**Methods:**

This was a phase I, open-label, 3 + 3 dose-escalation study conducted in two stages. In stage I, Japanese patients with solid tumors were administered ipatasertib 200, 400, or 600 mg/day for 21 days of a 28-day cycle. In stage II, Japanese patients with castration-resistant prostate cancer were administered ipatasertib 200 or 400 mg/day in combination with abiraterone and prednisolone in 28-day cycles. Dose-limiting toxicity (DLT) was assessed at each dose before enrolling patients at a higher dose; DLT was used to determine the maximum tolerated dose (MTD) and maximum administered dose (MAD). Pharmacokinetic parameters were assessed after a single dose and at steady state.

**Results:**

Fifteen patients were enrolled in Stage I and six in Stage II. The ipatasertib MTD was 600 mg as monotherapy and MAD was 400 mg in combination with abiraterone and prednisolone. Ipatasertib plasma exposure was dose proportional across the dose range, and was not markedly affected by concurrent administration of abiraterone plus prednisolone. Stable disease (SD) was observed in eight patients treated with ipatasertib monotherapy (53.3%); four patients had SD and one had complete response with ipatasertib plus abiraterone and prednisolone.

**Conclusions:**

Ipatasertib, at the monotherapy MTD of 600 mg/day and MAD of 400 mg/day in combination with abiraterone and prednisolone, was safe and tolerable in Japanese patients with solid tumors.

**Electronic supplementary material:**

The online version of this article (10.1007/s00280-019-03882-7) contains supplementary material, which is available to authorized users.

## Introduction

The phosphatidylinositol 3-kinase (PI3K)/Akt/mammalian target of rapamycin (mTOR) signaling pathway is a key regulator of cellular responses to stress [1]. The tumor microenvironment is inherently stressful, with poor oxygenation, low pH, and limited nutrient supply [1]. It is, therefore, unsurprising that this pathway plays a central role in the development and potentiation of cancer [1, 2]. Activation of this pathway by mutations of the *PIK3CA* gene or loss of tumor suppressor phosphatase and tensin homolog (PTEN) protein expression promotes tumor growth and proliferation [3, 4]. Serine/threonine kinase Akt (protein kinase B) plays an important role in the PI3K/Akt/mTOR pathway, and abnormally activated Akt is commonly seen in cancer [2, 5], including metastatic castration-resistant prostate cancer (mCRPC) [6, 7]. Furthermore, non-clinical data suggest that reciprocal crosstalk between the androgen receptor and PI3K/Akt/mTOR pathways is present in PTEN-loss mCRPC. Specifically, activation of the PI3K/Akt/mTOR pathway is associated with repressed androgen signaling, and inhibition of the PI3K/Akt/mTOR pathway restores androgen receptor signaling in PTEN-deficient prostate cells [8]. This suggests that combined inhibition of the androgen receptor and PI3K/Akt/mTOR pathways may result in measurable decline of tumor cell viability and more durable clinical benefit.

The central role of the PI3K/Akt/mTOR pathway in the oncogenic process has led to the development of cancer treatments targeting this pathway. For example, drugs that target the PI3K/Akt/mTOR pathway have shown activity in a range of cancers, including renal cell carcinoma [9] and triple-negative breast cancer (TNBC) [10], where conventional anti-cancer therapies have failed. However, most of the drugs that target PI3K/Akt/mTOR have shown limited activity as monotherapy, and there is greater potential for these drugs when administered in combination therapy [6, 11, 12].

Ipatasertib is a highly selective small-molecule inhibitor of Akt (Akt1, Akt2, and Akt3) [13–15], and is in development as a single agent and in combination with other therapies for the treatment of cancers in which activation of the PI3K/Akt/mTOR pathway is involved in tumor growth or therapeutic resistance [16, 17]. Results of a randomized, double-blind phase II study of ipatasertib in combination with abiraterone and prednisone/prednisolone showed trends towards improved radiographic progression-free survival (PFS) and overall survival (OS) compared with placebo in patients with mCRPC who had a PTEN loss [11]. The treatment was well tolerated [11]. Similarly, in patients with TNBC, the randomized, double-blind phase II study (LOTUS) reported longer PFS with the combination of ipatasertib plus paclitaxel than with placebo plus paclitaxel, indicating the benefits of ipatasertib in this patient population [18].

The current phase I dose-escalation study was undertaken to investigate the safety, tolerability, and pharmacokinetics of ipatasertib alone and in combination with abiraterone + prednisolone for Japanese patients with advanced or recurrent/refractory solid tumors.

## Materials and methods

### Study design

This was a phase I, open-label, multicenter, 3 + 3 dose-escalation study (JapicCTI-152,910) conducted at three centers in Japan. The study consisted of two stages. Stage I was designed to determine the maximum tolerated dose (MTD) and maximum administered dose (MAD) of ipatasertib monotherapy in Japanese patients with advanced or recurrent solid tumors, by investigating the safety, tolerability, and pharmacokinetics of ipatasertib in this population. Stage II determined the safety, tolerability, pharmacokinetics, and MTD/MAD of ipatasertib in combination with abiraterone and prednisolone in Japanese patients with CRPC.

The study protocol was approved by the institutional review boards of all participating centers and the study was conducted in accordance with the Declaration of Helsinki, Good Clinical Practice, and the Law for Ensuring the Quality, Efficacy, and Safety of Drugs and Medical Devices (paragraph 3 of article 14 and article 80–2).

All study participants provided written informed consent before entering the study.

### Patients

Patients were included in the study if they were aged ≥ 20 years with a histologically or cytologically confirmed, advanced or recurrent/refractory solid tumor (Stage I), or CRPC refractory to ≥ 1 type of hormone therapy with serum testosterone levels of < 50 ng/dL and who were not candidates for docetaxel or in whom docetaxel was ineffective (Stage II). In addition, patients were required to have an Eastern Cooperative Oncology Group performance status (ECOG PS) of 0 or 1; a life expectancy of ≥ 12 weeks after enrollment; lesion(s) that could be assessed by diagnostic imaging; major organ functioning within the required limits and sufficient cardiac function; a history of completing surgery, radiotherapy, chemotherapy, immunosuppressive therapy or treatment with other investigational drugs ≥ 4 weeks before the study; or blood transfusion/hematopoietic factor products, endocrine therapy or immunotherapy ≥ 2 weeks before the study.

Major exclusion criteria were hypersensitivity to hydroxypropyl methylcellulose (an excipient of ipatasertib); inability to take oral drugs or the presence of gastrointestinal issues that may interfere with drug absorption; meningeal or central nervous system (CNS) metastasis requiring treatment; previous adverse event (AE; grade ≥ 3) with an investigational product targeting Akt; diabetes mellitus requiring insulin; or an autoimmune disease or hypercalcemia requiring treatment. Additional exclusion criteria in Stage II were hypersensitivity to abiraterone or prednisolone, and a history of adrenal insufficiency or hyperaldosteronism. A complete list of inclusion and exclusion criteria is shown in Online Resource 1.

### Treatments

**Fig. 1:**
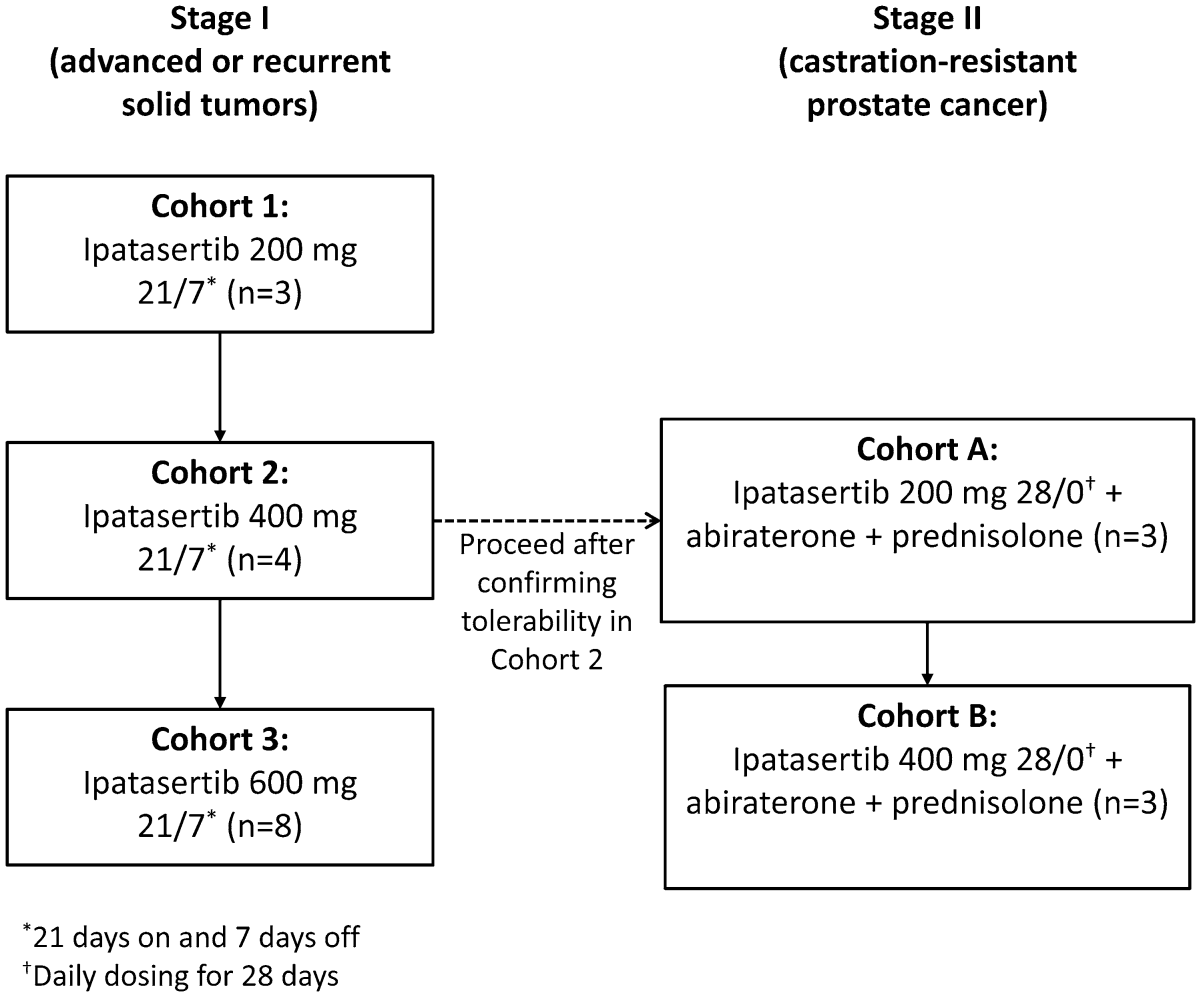
Study design

The study design and ipatasertib administration protocols are summarized in Fig. 1 and Online Resource 2, respectively.

In Stage I, patients received ipatasertib orally at escalating doses (200 mg, 400 mg, and 600 mg). First, they received a single dose on Day 1 followed by an off-treatment period. Subsequently, they received that dose of ipatasertib once daily for 21 days, followed by 7 days off, in 28-day cycles. The doses used were the same as the doses in the previous phase I study of ipatasertib [17].

In Stage II, patients received ipatasertib orally (200 mg and 400 mg once daily for 28 days), followed by abiraterone (1000 mg once daily) and prednisolone (5 mg twice daily). This was the same dose as used in the previous phase II study of ipatasertib [11]. The dose escalation strategy used for the two treatment stages is shown in Online Resource 3.

The treatments were continued until progressive disease (PD), dose-limiting toxicity (DLT), or withdrawal of informed consent.

Concomitant administration of the following agents was prohibited during the study: anti-tumor drugs, prophylactic treatments to prevent AEs including granulocyte colony-stimulating factors, St. John’s wort, grapefruit, long-term systemic corticosteroids (except prednisolone administered in Stage II), other investigational or unapproved drugs, and drugs that prolong QT interval (Stage I).

### Study outcomes

The primary objectives of this study were to determine the safety, tolerability, and pharmacokinetics of ipatasertib alone and in combination with abiraterone and prednisolone. Safety and tolerability were assessed by the occurrence of AEs and DLTs, and DLT was used to determine MTD and MAD. The severity of AEs was graded according to National Cancer Institute Common Terminology Criteria for Adverse Events (NCI CTCAE), version 4.03 [19].

DLTs were defined as the occurrence of AEs during the evaluation period for which a causal relationship with ipatasertib could not be ruled out, and which met the treatment discontinuation criteria or required drug suspension during the evaluation period. The DLT observation period for Stage I was from Day 1 of Cycle 0 to before administration on Day 1 of Cycle 2, and for Stage II, it was from Day 1 of Cycle 1 to before administration on Day 1 of Cycle 2. A complete list of potential DLTs considered in the study is included in Online Resource 4 and contains grade 4 neutropenia for ≥ 5 days; febrile neutropenia; grade 3 thrombocytopenia requiring platelet transfusions or grade 4 thrombocytopenia; grade ≥ 4 anemia; grade ≥ 3 non-hematologic toxicity (excluding transient electrolyte abnormalities). The occurrence of a DLT at any given dose determined whether or not investigators would proceed to the next dose cohort. DLTs were used to determine the MTD of ipatasertib alone and in combination with abiraterone and prednisolone, defined as the highest dose at which < 33% of patients experienced a DLT. The MAD of ipatasertib alone was determined to be 600 mg if no patients experienced a DLT in Cohort 3, and that of ipatasertib in combination with abiraterone and prednisolone was determined to be 400 mg if no patients experienced a DLT in Cohort B.

Pharmacokinetic parameters (*t*_max_, *C*_max_, AUC_0–24_, *t*_1/2_) for ipatasertib and its metabolite (G-037720) following a single dose (Stage I: Cycle 0, Day 1; Stage II: Cycle 1, Day 1) and repeated doses (Stage I: Cycle 1, Day 8; Stage II: Cycle 1, Day 15) were calculated using plasma drug concentration–time data. Serial blood samples were taken for 72 h after a single dose in Cycle 0 and on Day 8 in Cycle 1 during Stage I, and on Day 1 and Day 15 of Cycle 1 in Stage II (Online Resource 5).

Concentrations of ipatasertib and G-037720 were determined using a validated liquid chromatography–tandem mass spectrometry analytical procedure, with a lower limit of quantification of 0.500 ng/mL for both ipatasertib and G-037720. The accumulation ratio was calculated using the formula: [AUC_0–24_ at steady state]/[AUC_0–24_ following single dose].

The secondary objective of the study was to determine the preliminary efficacy of ipatasertib in both stages of the study. All tumor lesions were assessed using the Response Evaluation Criteria in Solid Tumors (RECIST) version 1.1 [20].

An exploratory objective of this study was to determine the relationship between tumor response and PTEN expression, and PI3K pathway gene mutation and amplification. *PIK3CA* and *Akt1* mutation/amplification were detected in tumor tissue samples collected prior to study entry (archival samples) using a Semiconductor DNA sequencer and Ion AmpliSeq™ Cancer Hotspot Panel, version 2 (Thermo Fisher Scientific; Waltham, MA, USA). Copy number variations (CNVs) were detected in the *Akt1* and *PIK3CA* genes, and were reported if CNV confidence value was ≥ 20. Single nucleotide polymorphisms (SNPs) were reported if they had a frequency of ≥ 1, coverage of ≥ 500, and were located in a known hotspot (allele source). PTEN expression was analyzed in formalin-fixed, paraffin-embedded tissue samples by immunohistochemistry (IHC) using the VENTANA OptiView DAB IHC Detection Kit on the automated BenchMark ULTRA platform (Ventana Medical Systems; Tucson, AZ, USA) with the PTEN (SP218) rabbit monoclonal antibody assay (Spring Biosciences; Pleasanton, CA, USA) [21]. Once acceptable internal controls had been met, PTEN was considered to be intact if the specimen contained > 50% of viable malignant cells with any specific cytoplasmic stain intensity, and was considered to be lost if ≥ 50% of viable malignant cells had no specific cytoplasmic staining [21]. Nuclear staining of viable malignant cells was disregarded.

### Statistical analysis

The planned sample size for the study was 15–30 patients in total, 9–18 patients in Stage I (3–6 per cohort) and an additional 6–12 patients in Stage II (3–6 per cohort). The safety analysis set included all patients who received ≥ 1 dose of the study drug, and the DLT population included all patients from the safety analysis set who were evaluable for DLTs. The full analysis set included all patients who received ≥ 1 dose of study drug and who subsequently underwent ≥ 1 efficacy assessment.

The calculation of pharmacokinetic parameters was performed using WinNonlin Ver 6.4 (Pharsight Corporation, NC, USA), and data aggregation was performed using SAS, version 9 (SAS Institute Inc., NC, USA).

## Results

### Patients

The study was conducted at three centers between 29 May 2015 and 24 August 2017. Overall, 21 patients were enrolled, 15 in Stage I and 6 in Stage II (Table 1). Patients enrolled in Stage I had a median age of 58.0 years (range 35–76) and were mostly male (53.3%); the majority of patients (80%) had an ECOG PS of 0 (Table 1). In Stage II, patients had a median age of 70.5 years (range 45–77); all patients were male (100%) and most (83.3%) had an ECOG PS of 0 (Table 1). All patients in Stage II had received prior systemic therapies, including chemotherapy in four patients (66.7%) and abiraterone or enzalutamide in five (83.3%).

**Table 1:** Baseline characteristics of patients included in the study (*N* = 21)

	Stage I				Stage II		
	Ipatasertib 200 mg (*n* = 3)	Ipatasertib 400 mg (*n* = 4)	Ipatasertib 600 mg (*n* = 8)	Total (*n* = 15)	Ipatasertib 200 mg + ABI + PRE (*n* = 3)	Ipatasertib 400 mg + ABI + PRE (*n* = 3)	Total (*n* = 6)
Sex, *n* (%)							
Male	1 (33.3)	1 (25.0)	6 (75.0)	8 (53.3)	3 (100.0)	3 (100.0)	6 (100.0)
Age^a^, years	37.0 (35–58)	59.5 (54–68)	66.0 (49–76)	58.0 (35–76)	71.0 (62–74)	70.0 (45–77)	70.5 (45–77)
Weight^a^, kg	49.00 (47.2–57.1)	58.85 (52.3–68.9)	59.10 (47.3–73.9)	58.05 (47.2–73.9)	62.90 (59.3–83.8)	68.80 (68.8–84.2)	68.80 (59.3–84.2)
ECOG PS, *n* (%)							
0	2 (66.7)	3 (75.0)	7 (87.5)	12 (80.0)	3 (100.0)	2 (66.7)	5 (83.3)
1	1 (33.3)	1 (25.0)	1 (12.5)	3 (20.0)	0 (0.0)	1 (33.3)	1 (16.7)
Number of prior systemic therapies, *n* (%)							
2	0	1 (25.0)	1 (12.5)	2 (13.3)	0	0	0
≥ 3	3 (100.0)	3 (75.0)	7 (87.5)	13 (86.7)	3 (100.0)	3 (100.0)	6 (100.0)
PSA^a^, μg/L	–	–	–	–	5.3 (4.5–202.2)	96.5 (43.6–236.5)	70.1 (4.5–236.5)
Type of cancer, *n* (%)							
Bladder	0	0	3 (37.5)*	3 (20.0)	0	0	0
Cervical	0	0	2 (25.0)	2 (13.3)	0	0	0
Colorectal	0	0	1 (12.5)	1 (6.7)	0	0	0
CRPC	0	0	0	0	3 (100.0)	3 (100.0)	6 (100.0)
Duodenum papilla	0	0	1 (12.5)	1 (6.7)	0	0	0
Gastric	1 (33.3)	0	0	1 (6.7)	0	0	0
GIST	0	0	1 (12.5)	1 (6.7)	0	0	0
Liver	1 (33.3)	0	1 (12.5)*	2 (13.3)	0	0	0
Ovarian	1 (33.3)	1 (25.0)	0	2 (13.3)	0	0	0
Peritoneal	0	1 (25.0)	0	1 (6.7)	0	0	0
Ureteral	0	1 (25.0)	0	1 (6.7)	0	0	0
Unknown	0	1 (25.0)	0	1 (6.7)	0	0	0
Cancer histology, n (%)							
Adenocarcinoma	2 (66.7)	3 (75.0)	2 (25.0)	7 (46.7)	3 (100.0)	3 (100.0)	6 (100.0)
GIST	0	0	1 (12.5)	1 (6.7)	0	0	0
HCC	1 (33.3)	0	1 (12.5)*	2 (13.3)	0	0	0
SCC	0	0	2 (25.0)	2 (13.3)	0	0	0
UC	0	1 (25.0)	3 (37.5)*	4 (26.7)	0	0	0

*ABI* abiraterone, *CRPC* castration-resistant prostate cancer, *ECOG PS* Eastern Cooperative Oncology Group performance status, *GIST* gastrointestinal stromal tumor, *HCC* hepatocellular carcinoma, *PRE* prednisolone, *PSA* prostate-specific antigen, *SCC* squamous cell carcinoma, *UC* urothelial carcinoma

*One patient had bladder and liver cancer (histologist UC and HCC, respectively)

^a^Median (range)

### Safety

Ipatasertib was well tolerated at doses up to 600 mg as monotherapy in Stage I and up to 400 mg as combination therapy in Stage II. At least one AE was experienced by all patients, most commonly diarrhea and nausea (Table 2). Grade 3 AEs developed in four patients treated with ipatasertib 600 mg during Stage I. These events were nausea (*n* = 2), hyperglycemia (*n* = 2), diarrhea (*n* = 1), and colitis/dehydration (*n* = 1). During Stage I, serious AEs (SAEs) were reported in one patient who developed grade 3 colitis that was considered related to study drug, accompanied by grade 3 dehydration that was considered unrelated to study drug; no SAEs occurred in Stage II of the study. The patient made a complete recovery after treatment discontinuation. Two patients developed grade 3 AEs during Stage II while receiving ipatasertib 400 mg in combination therapy; these events were urticaria (*n* = 1) and anemia (*n* = 1). No grade 4 AEs or deaths occurred during either of the two stages.

**Table 2:** Adverse events in Stage I and Stage II of the study (*N* = 21)

AEs, *n* (%)	Stage I				Stage II		
	Ipatasertib 200 mg (*n* = 3)	Ipatasertib 400 mg (*n* = 4)	Ipatasertib 600 mg (*n *= 8)	Total (*n* = 15)	Ipatasertib 200 mg + ABI + PRE (*n* = 3)	Ipatasertib 400 mg + ABI + PRE (*n* = 3)	Total (*n* = 6)
Any	3	4	8	15 (100)	3	3	6 (100)
AEs reported in ≥ 2 patients							
Diarrhea	–	3	7	10 (66.7)	2	3	5 (83.3)
Nausea	1	2	7	10 (66.7)	3	3	6 (100)
Decreased appetite	–	2	5	7 (46.7)	–	1	1 (16.7)
Vomiting	–	1	4	5 (33.3)	1	1	2 (33.3)
Fatigue	1	2	2	5 (33.3)	–	1	1 (16.7)
Hyperglycemia	–	–	2	2 (13.3)	–	–	–
AST increased	–	–	2	2 (13.3)	–	–	–
ALT increased	–	–	2	2 (13.3)	–	–	–
Blood insulin increased	–	–	2	2 (13.3)	–	–	–
Blood creatinine increased	–	1	1	2 (13.3)	–	–	–
Glucose urine present	–	–	2	2 (13.3)	–	–	–
Rash	1	–	1	2 (13.3)	–	1	1 (16.7)
Back pain	1	1	–	2 (13.3)	–	–	–
Diabetes mellitus	–	–	–	–	2	–	2 (33.3)
Dysgeusia	–	–	1	1 (6.7)	–	2	2 (33.3)
Dizziness	–	–	–	–	–	2	2 (33.3)

*ABI* abiraterone, *ALT* alanine aminotransferase, *AST* aspartate aminotransferase, *PRE* prednisolone

In Stage I, 12 of 15 patients were evaluated for DLT; 3 patients (400 mg, *n* = 1; 600 mg, *n* = 2) were not evaluable because they discontinued the study before the end of the evaluation period for reasons other than AEs (patient decision). No DLTs were reported with ipatasertib 200 mg or 400 mg, and one patient on ipatasertib 600 mg experienced grade 3 nausea, which required drug withdrawal for more than 6 days, during the DLT observation period. The MTD for ipatasertib was 600 mg/day for 21 days of a 28-day cycle.

No DLTs developed during Stage II of the study. The MAD for ipatasertib was 400 mg/day when used in combination with abiraterone and prednisolone in the 28-day cycle schedule.

### Pharmacokinetics

The pharmacokinetic study population in Stage I consisted of 14 patients. Data from one patient in Stage I (200 mg) were excluded from the pharmacokinetic analysis because this patient had a history of total surgical gastrectomy and lower esophagectomy, which could affect drug absorption.

**Fig. 2:**
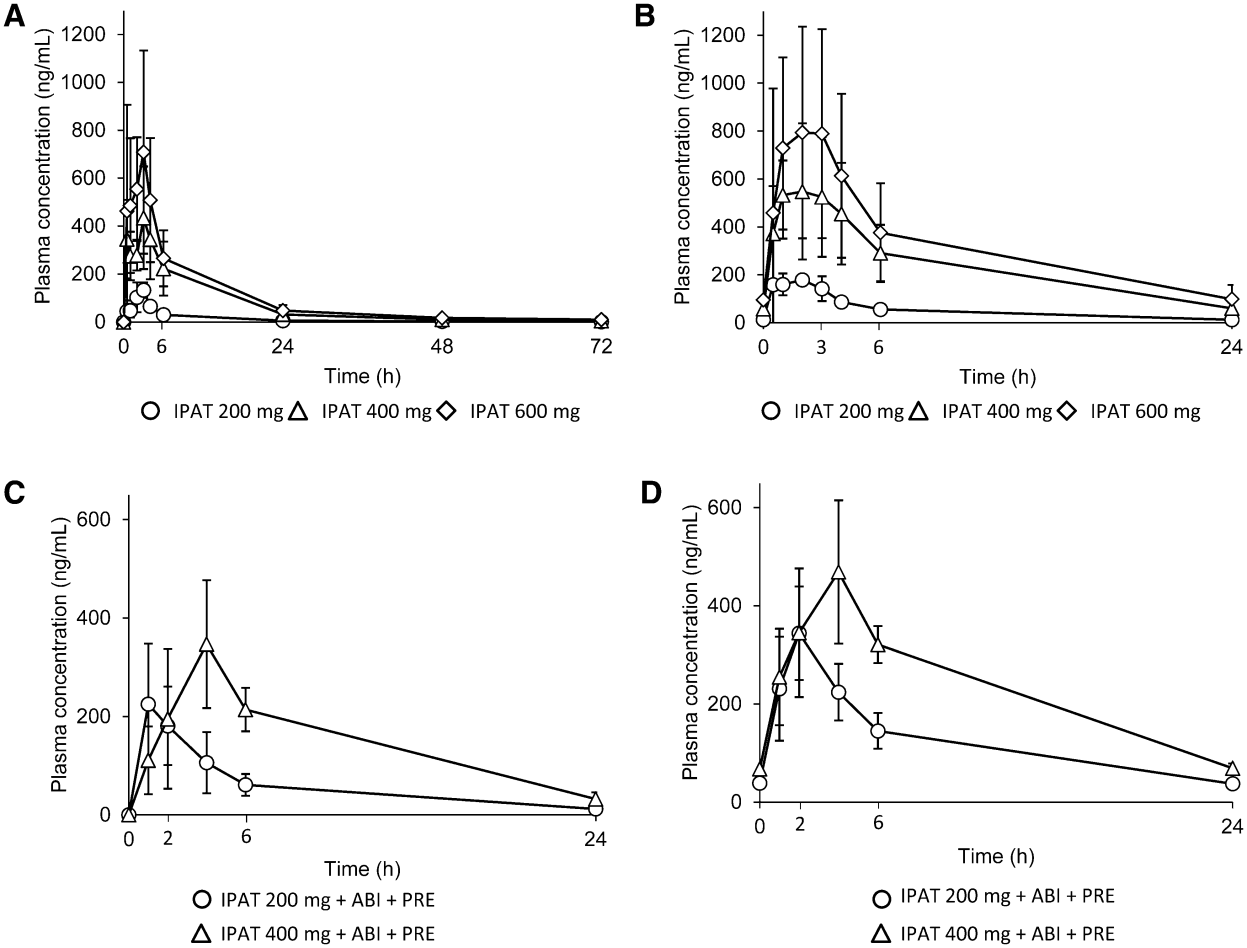
Mean (standard deviation) plasma concentration of ipatasertib at steady state after single and repeated doses. **a** Stage I, single dose (Cycle 0, Day 1); **b** Stage I, repeated doses (Cycle 1, Day 8); **c** Stage II, single dose (Cycle 1, Day 1); and **d** Stage II repeated doses (Cycle 1, Day 15). ABI, abiraterone; IPAT, ipatasertib; PRE, prednisolone

Ipatasertib as a single agent was rapidly absorbed after oral administration. The *t*_max_ was reached at a median of 2.53–3.03 h after the first administration of ipatasertib at a dose of 200–600 mg. The geometric mean *t*_1/2_ was between 18.8 and 24.3 h at these doses (Table 3). The plasma ipatasertib concentration reached steady state within 7 days after daily administration, with an accumulation ratio between 1.38 and 1.82 (Table 3). The plasma concentrations of ipatasertib increased proportionally with dose escalation in the dose range of 200–600 mg (Fig. 2a, b).

**Table 3:** Single dose and steady-state pharmacokinetic parameters of ipatasertib during the study

Single dose pharmacokinetics	Stage I			Stage II	
	Ipatasertib 200 mg	Ipatasertib 400 mg	Ipatasertib 600 mg	Ipatasertib 200 mg	Ipatasertib 400 mg
	Cycle 0, Day 1			Cycle 1, Day 1	
	(*n* = 2)	(*n* = 4)	(*n* = 8)	(*n* = 3)	(*n* = 3)
*C*_max_^a^, ng/mL	151 (3.75)	456 (36.6)	953 (36.0)	214 (49.9)	328 (46.0)
*T*_max_^b^, h	2.53 (1.98–3.08)	3.03 (1.02–4.07)	2.57 (0.52–4.00)	0.97 (0.95–3.98)	3.97 (3.90–4.02)
*t*_1/2_^c^, h	24.3 (23.7–24.8)	18.8 (17.0–21.1)	21.5 (16.2–33.9)	7.34 (7.23–7.45)	NC
AUC_0–24_^a^, h ng/mL	805 (30.7)	4010 (38.6)	5930 (33.1)	1250 (36.4)	2940 (29.6)

Steady-state pharmacokinetics	Stage I			Stage II	
	Ipatasertib 200 mg	Ipatasertib 400 mg	Ipatasertib 600 mg	Ipatasertib 200 mg	Ipatasertib 400 mg
	Cycle 1, Day 8			Cycle 1, Day 15	
	(*n* = 2)	(*n* = 4)	(*n* = 7)	(*n* = 3)	(*n* = 3)
*C*_max_^a^, ng/mL	186 (4.17)	579 (43.1)	973 (57.4)	334 (31.3)	452 (35.0)
*T*_max_^b^, h	1.46 (0.97–1.95)	1.48 (0.93–4.00)	1.97 (0.47–3.03)	1.98 (1.95–2.02)	3.97 (3.87–4.03)
*t*_1/2_^c^, h	7.69 (7.49–7.90)	7.20 (7.03–7.32)	8.06 (6.50–10.30)	8.29 (7.62–8.84)	NC
AUC_0–24_^a^, h ng/mL	1210 (23.5)	4870 (43.1)	6510 (57.6)	2710 (28.7)	4970 (17.8)
Accumulation ratio	1.82 (3.58)	1.43 (9.41)	1.38 (51.7)	2.16 (10.0)	1.69 (11.8)

*AUC*_*0–24*_ Area under concentration–time curve from 0 to 24 h, *NC* not calculated

^a^Geometric mean (% CV)

^b^Median (range)

^c^Geometric mean (range)

G-037720 was detected in plasma soon after the administration of a single dose of ipatasertib. Its median *t*_max_ was 3.00–3.05 h, and geometric mean *t*_1/2_ was 21.3–29.7 h after administration of ipatasertib 200–600 mg (Stage I, Cycle 0, Day 1). G-037720 was considered to be the main metabolite of ipatasertib, since the geometric mean of metabolite/parent (M/P) ratio of AUC_0–inf_ after single administration of 200–600 mg ipatasertib was 0.426–0.884.

Abiraterone and prednisolone did not markedly affect the plasma concentration profile of ipatasertib. The plasma concentrations of ipatasertib increased with dose escalation (single and repeated doses; Fig. 2c, d). The geometric mean AUC_0–24_ following repeated doses of ipatasertib 400 mg plus abiraterone and prednisolone (4970 h ng/mL, GCV 17.8%; Stage II, Cycle 1, Day 15) was comparable to that observed following repeated doses of ipatasertib 400 mg as a single agent (4870 h ng/mL, GCV 43.1%; Stage I, Cycle 1, Day 8). However, the AUC_0–24_ for G-037720 was approximately twofold higher in patients receiving multiple doses of ipatasertib 400 mg plus abiraterone and prednisolone (Stage II, Cycle 1, Day 15) compared with patients receiving ipatasertib 400 mg as a single agent [Stage I, Cycle 1, Day 8; 4540 (33.9) vs. 2230 (38.0) h ng/mL (GCV%)].

### Efficacy

**Fig. 3:**
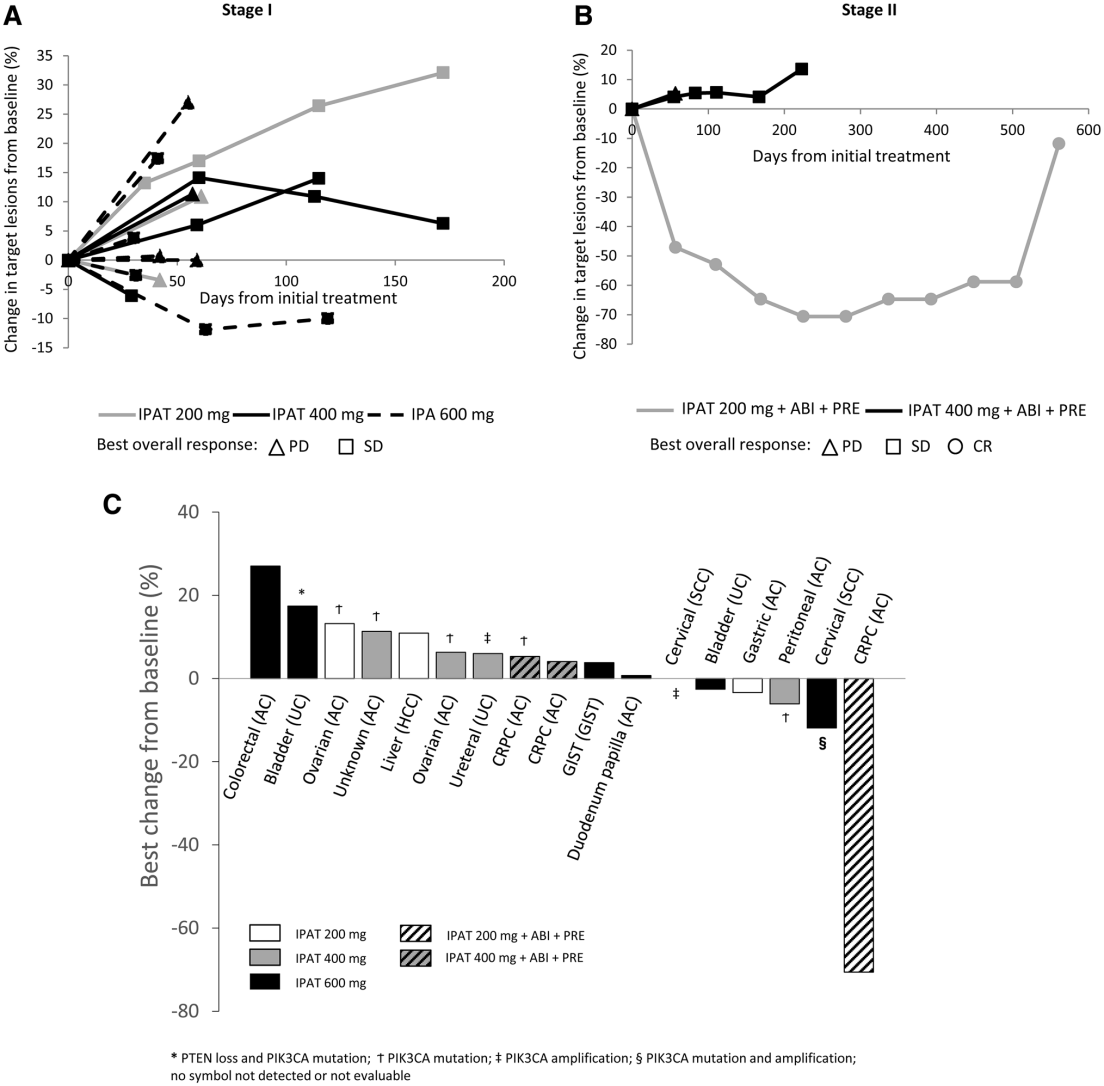
Percentage change from baseline in tumor lesions during **a** Stage I (*n* = 14); **b** Stage II (*n* = 3); and **c** best percentage change from baseline in target lesions and *PIK3CA* mutation/amplification and PTEN loss. *ABI* abiraterone, *AC* adenocarcinoma, *CR* complete response, *CRPC* castration-resistant prostate cancer, *GIST* gastrointestinal stromal tumor, *HCC* hepatocellular carcinoma, *IPAT* ipatasertib, *PD* progressive disease, *PRE* prednisolone, *SCC* squamous cell carcinoma, *SD* stable disease, *UC* urothelial carcinoma

During Stage I, efficacy was evaluated in 14 of 15 patients treated with ipatasertib. One patient discontinued the treatment before the post-treatment tumor assessment and was excluded from the efficacy evaluation. The best overall response was stable disease (SD) in eight patients and PD in six patients. The percentage change from baseline in target lesions is shown in Fig. 3a.

All six patients treated with ipatasertib during Stage II who were evaluable had a treatment history of more than four regimens for CRPC. One of these patients had a CR, four patients had SD, and one had PD. The percentage change from baseline in target lesions of three patients who had measurable lesions at screening is shown in Fig. 3b. Three patients were able to continue treatment for six cycles or more, despite the fact that two of them had a history of abiraterone and enzalutamide treatment.

### Gene alteration status

A total of 15 tumor samples were evaluated for PTEN, *PIK3CA,* and *Akt1*. PTEN status was evaluable in seven patients, one of whom had PTEN loss with SD. *PIK3CA* mutations were detected in eight patients, five of whom had SD and one of whom was not evaluable. Tumor shrinkage (− 11.9%, − 6.1%) was observed in two patients (one with cervical cancer; one with peritoneal cancer) who had *PIK3CA* mutation in the helical domain (E542K or E545K) and/or amplification (Fig. 3c). *PIK3CA* amplification was detected in three patients, while *Akt1* mutation and amplification were not detected.

## Discussion

This 3 + 3 dose-escalation phase I study showed that ipatasertib was well tolerated, with a favorable safety profile when administered either alone (MTD, 600 mg/day) or in combination with abiraterone and prednisolone (MAD, 400 mg/day). The data also show that ipatasertib was rapidly absorbed after oral administration, and its plasma concentration profile is unaffected by concomitant administration of abiraterone and prednisolone. When used as monotherapy in patients with solid tumors, the best overall response with ipatasertib was SD in eight patients, while the best response with ipatasertib plus abiraterone and prednisolone was CR in one patient and SD in four patients.

During the present study, the most common AEs observed with ipatasertib monotherapy were diarrhea, nausea, decreased appetite, fatigue, and vomiting, and the most common AEs with combination therapy were nausea and diarrhea. These events were mostly grade 1 or 2 in severity. Grade 3 events developed in four patients with ipatasertib monotherapy (nausea, hyperglycemia, diarrhea, and colitis/dehydration) and in two patients during combination therapy (anemia and urticaria). No patients developed grade 4 or 5 AEs during ipatasertib treatment as either monotherapy or in combination. The safety profile of ipatasertib as monotherapy or combination therapy in this study was consistent with what is expected of agents targeting the PI3K/Akt/mTOR pathway and with the safety profile of ipatasertib observed in non-Japanese patients. The phase I and II studies with ipatasertib in non-Japanese patients also reported diarrhea, nausea, and hyperglycemia as common AEs [11, 17].

The AUC_0–24_ and *C*_max_ for ipatasertib monotherapy were found to be dose dependent in the present study. The mean AUC_0–24_ of ipatasertib at steady state was approximately 0.7- to 1.5-fold of that reported in previous phase I study of ipatasertib [17]. Although the mean plasma exposures were higher, the plasma exposures in individual patients showed significant overlap, and the data may be confounded by the small number of patients evaluated for this comparison.

Combining ipatasertib with abiraterone and prednisolone in Stage II of the present study did not majorly affect the plasma concentration profile of ipatasertib. Although a twofold increase in AUC_0–24_ of the main metabolite of ipatasertib (G-037,720) was observed after repeated administration of the combination compared with ipatasertib monotherapy, G-037,720 is less active compared with ipatasertib and is expected to have limited anticancer activity. The exact reason for this increase in AUC_0–24_ is unknown. Although the pharmacokinetics of abiraterone in combination with ipatasertib were not analyzed in this study, they were assessed in a previous study [11] and the AUC and *C*_max_ of abiraterone were shown to be similar to that of abiraterone monotherapy (data not published).

PTEN loss and *PIK3CA/AKT1* mutation/amplification have been studied as potential biomarkers for ipatasertib response in combination therapy. The A.MARTIN study in patients with mCRPC who were treated with ipatasertib plus abiraterone and prednisone/prednisolone showed that the combination increased radiographic PFS in patients with PTEN loss, indicating that PTEN loss may be a predictive biomarker of response [22].

The LOTUS study reported that ipatasertib in combination with paclitaxel improved PFS in patients with *PIK3CA/Akt1/PTEN*-altered TNBC, suggesting that *PIK3CA/Akt1/PTEN* alterations can also be biomarkers of response to ipatasertib in patients with breast cancer [18]. In the present study, tumor shrinkage was observed in two patients with *PIK3CA* mutation in the helical domain, which is a hotspot for *PIK3CA* mutations [23, 24]. However, none of the tumor samples in our cohort had *Akt1* mutations or amplifications.

The genetic basis of prostate cancer is complex and our understanding is continually evolving [25]. A recent systematic review noted that, in addition to genetic alterations in the PIK3CA/Akt1/PTEN pathway, prognosis in prostate cancer may be associated with alterations in genes controlling DNA methylation, such as those at the glutathione S-transferase pi (*GSTP1*) and the familial protein 1 isoform A (*RASSF1A*) loci, and in androgen regulation, such as *TMPRSS2* and *ERG* [25]. In addition, the most common genetic mechanism of PTEN loss in prostate cancer is deletion of the *10q23* locus, whereas inactivating mutations predominate in other cancers [26]. As well as inactivating PTEN, *10q23* loss may impair the expression of surrounding genes (including tumor suppressors), which may also affect outcomes and treatment responses in prostate cancer [26]. The results of large-scale studies, such as phase III studies, are needed to clarify which molecular biomarkers can act as prognostic indicators in patients receiving ipatasertib.

The main limitation of the present study was that potential biomarkers of tumor response to ipatasertib could not be determined due to the small patient population. In addition, because this trial focused on a small group of Japanese patients, it is unclear that the safety results of ipatasertib from this trial can be generalized to other ethnic groups. Large trials are required to confirm the safety and efficacy of ipatasertib, and to clarify the prognostic significance of genetic alternations in patients who are receiving ipatasertib.

As noted earlier, the number of patients who underwent genetic analysis in this study was too small to detect a relationship between the tumor response and PTEN expression or mutation/amplification of *PIK3CA* and *Akt1*. Another potential issue is the use of archival samples because these samples may not reflect the gene profile of tumor tissues at the time that response was evaluated. Among available samples in the current study, the rate of PTEN loss was 14%, which is lower than in other reports in men with CRPC (~ 40%) [27, 28]. One reason may be that genetic alterations in the PIK3CA/Akt1/PTEN pathway are less frequent among Asian men with prostate cancer than among Caucasian men [29, 30]. Therefore, the prognostic value of these molecular alterations probably varies by ethnicity.

In conclusion, ipatasertib as a monotherapy (MTD, 600 mg/day) and in combination with abiraterone plus prednisolone (MAD, 400 mg/day) was safe and well tolerated in Japanese patients with advanced or recurrent refractory solid tumors. Currently, there are two ongoing global phase III studies for ipatasertib that include Japanese patients. These are examining ipatasertib in combination with abiraterone plus prednisone/prednisolone for patients with mCRPC (NCT03072238) [31] and ipatasertib in combination with paclitaxel for metastatic TNBC/hormone receptor-positive breast cancer (NCT03337724) [32].

## Electronic supplementary material

Below is the link to the electronic supplementary material.

Supplementary material 1 (DOCX 18 kb)

Supplementary material 2 (DOCX 68 kb)

Supplementary material 3 (DOCX 46 kb)

Supplementary material 4 (DOCX 15 kb)

Supplementary material 5 (DOCX 15 kb)
