# Supplementary material for: Phase I study of ipatasertib as a single agent and in combination with abiraterone plus prednisolone in Japanese patients with advanced solid tumors
Source: Cancer Chemother Pharmacol. 2019 Jun 21;84(2):393–404. doi: 10.1007/s00280-019-03882-7 (PMC6647215; doi:10.1007/s00280-019-03882-7)
Supplement: Supplementary file 1 — Supplementary material 1 (DOCX 18 kb) [file 280_2019_3882_MOESM1_ESM.docx]

**Online Resource 1.** Complete inclusion and exclusion criteria

| **Inclusion** | **Exclusion** |
| --- | --- |
| Common to stages I and II  Provided written informed consent in person after having received a thorough explanation of the study;  Aged ≥20 years at the time of informed consent;  ECOG Performance Status of 0 or 1;  Life expectancy of ≥12 weeks after enrollment;  Evaluable lesion by imaging;  Major organ function meeting the criteria below within 2 weeks before enrollment. If multiple test results were available for this period, those obtained closest to enrollment were used.  Neutrophil count: ≥1,500/μL  Platelet count: ≥75,000/μL  Hemoglobin: ≥9.0 g/dL  Serum albumin: ≥3.0 g/dL  Total bilirubin: ≤1.5 times the upper limit of the laboratory reference range;  AST and ALT: ≤3 times the upper limit of the laboratory reference range;  If there was a tumor lesion on the liver, ≤5 times the upper limit of the laboratory reference range;  ALP: ≤2.5 times the upper limit of the laboratory reference range;  If there was a tumor lesion on the liver or a bone, ≤5 times the upper limit of the laboratory reference range;  Fasting blood glucose: ≤140 mg/dL  HbA1c (NGSP value): ≤6.5%  Total cholesterol: ≤300 mg/dL  Triglycerides: ≤300 mg/dL  Serum creatinine: ≤1.5 times the upper limit of the laboratory reference range;  PT-INR and APTT: ≤1.5 times the upper limit of the laboratory reference range;  Sufficient cardiac function (left ventricular ejection fraction calculated by echocardiography ≥50%);  Must have completed the following previous treatments or interventions by at least the following time before enrollment:  Surgery^a^, radiotherapy^b^: 4 weeks  Chemotherapy (including molecular-targeted drugs): 4 weeks  Blood transfusion, hematopoietic factor products: 2 weeks  Endocrine therapy^c^, immunotherapy: 2 weeks  Immunosuppressive therapy^d^: 4 weeks  Other investigational medicinal products: 4 weeks  Stage I only  Histologically or cytologically confirmed solid tumor;  Advanced or recurrent cancer that was refractory to the standard of care or for which no standard of care exists.  Stage II only  Histologically or cytologically confirmed castration-resistant prostate cancer;  Refractory to at least 1 type of hormone therapy for castration-resistant prostate cancer, and docetaxel treatment was either ineffective or not applicable. In addition, serum testosterone was <50 ng/dL (or 1.7 nM). | Common stages I and II  History of hypersensitivity to an excipient of ipatasertib (hydroxypropyl methylcellulose);  Inability to take oral drugs or gastrointestinal dysfunction or inflammatory bowel disease that would interfere with drug absorption;  Meningeal metastasis or metastasis to the central nervous system requiring treatment or accompanied by symptoms;  Pleural effusion, cardiac effusion, or ascites that required fluid drainage; however, patients for whom at least 2 weeks had passed since fluid drainage and who had no exacerbation at the time of enrollment were eligible;  Ongoing adverse reaction to a previous treatment with NCI CTCAE grade ≥2 severity (Version 4.03) (<9.0 g/dL for hemoglobin); however, alopecia was permissible;  Previously experienced an AE of NCI CTCAE (Version 4.03) grade ≥3 severity following administration of an investigational product targeting Akt;  Active infection requiring systemic administration of an antibiotic, antifungal, or antiviral, etc.;  Autoimmune disease requiring treatment;  Hypercalcemia requiring treatment with a bisphosphonate; however, use of a bisphosphonate for any other reason (e.g., bone metastasis or osteoporosis) was permissible;  Concurrent or previous clinically significant liver disease (e.g., viral, alcoholic, or other hepatitis or hepatic cirrhosis);  Positive test result for HIV antibodies, HBs antigen, HBs antibodies, HBc antibodies, or HCV antibodies (ineligible even if HCV-RNA-negative); however, patients who were only positive for HBs antibody and whose vaccination history was known, and patients who were only positive for HBs antibody and/or HBc antibody and negative for HBV-DNA, were also eligible for enrollment;  Concurrent or previous significant lung disease;  Concurrent cerebrovascular disorder (e.g., subarachnoid hemorrhage, cerebral infarction, or transient cerebral ischemic attack) with symptoms, or a history thereof within 6 months before enrollment;  Concurrent NYHA Class ≥II congestive cardiac failure, myocardial infarction, or unstable angina, or a history thereof within 6 months before enrollment;  History of ventricular arrhythmia within 6 months before enrollment, or arrhythmia requiring treatment, or QTc interval exceeding 480 ms;  Uncontrollable hypertension (SBP ≥140 mmHg, or DBP ≥90 mmHg);  Type 1 or Type 2 diabetes mellitus requiring treatment with insulin. Patients with diabetes mellitus requiring treatment with an oral drug other than insulin were eligible if their condition was stable over the 2 weeks before the start of treatment;  Men or women of childbearing potential (Stage 1 only) who were not willing to use an appropriate method of contraception during the treatment period and for 6 months after the last dose of the study drug;  Any other reason the investigator deemed a patient unsuitable for study participation.  Stage I only  Women who were pregnant or lactating (however, lactating women who were willing to stop nursing during the treatment period and for 6 months after the last dose of the investigational product were eligible.);  Women with a positive pregnancy test result (pregnancy testing was performed in women of childbearing potential who had menstruated within 12 months before enrollment. However, pregnancy testing was also performed in women who had not menstruated in the last 12 months if pregnancy could not be ruled out due to chemical menopause or other reason).  Stage II only  History of hypersensitivity to any of the drugs included in the concomitant therapy;  History of adrenal insufficiency or hyperaldosteronism. |

^a^Major surgery, such as that requiring thoracotomy or laparotomy, or surgery involving organ resection using a laparoscope. After an intervention involving an incision (including port implantation) that was not major surgery, enrollment was possible if the patient had recovered to an extent that it was judged that there was no risk of hemorrhage as of the time of enrollment

^b^After palliative radiotherapy to alleviate symptoms or gamma knife to treat brain micrometastasis: 2 weeks

^c^Other than hormone replacement therapy, oral contraceptives, and GnRH agonist/antagonist therapy for prostate cancer

^d^This included cyclophosphamide, azathioprine, methotrexate, and thalidomide. However, local or temporary corticosteroid use of ≤20 mg/day prednisolone or equivalent was permissible

AE, adverse event; ALP, alkaline phosphatase; ALT, alanine aminotransferase; APTT, activated partial thromboplastin time; AST, aspartate aminotransferase; DBP, diastolic blood pressure; DNA, deoxyribonucleic acid; ECOG, Eastern Cooperative Oncology Group; GnRH, gonadotrophin releasing hormone; HbA1c, glycosylated hemoglobin; HBc, hepatitis B virus core; HBs, hepatitis B virus surface; HCV, hepatitis C virus; HIV, human immunodeficiency virus; NCI CTCAE, National Cancer Institute Common Terminology Criteria for Adverse Events; NGSP, National Glycohemoglobin Standardization Program; NYHA, New York Heart Association; PT-INR, prothrombin time-international normalized ratio; RNA, ribonucleic acid; SBP, systolic blood pressure.
