# Supplementary figures and images for: Phase I study of ipatasertib as a single agent and in combination with abiraterone plus prednisolone in Japanese patients with advanced solid tumors
Source: Cancer Chemother Pharmacol. 2019 Jun 21;84(2):393–404. doi: 10.1007/s00280-019-03882-7 (PMC6647215; doi:10.1007/s00280-019-03882-7)

**Online Resource 2.** Treatment procedure. D, day; DLT, dose-limiting toxicity.


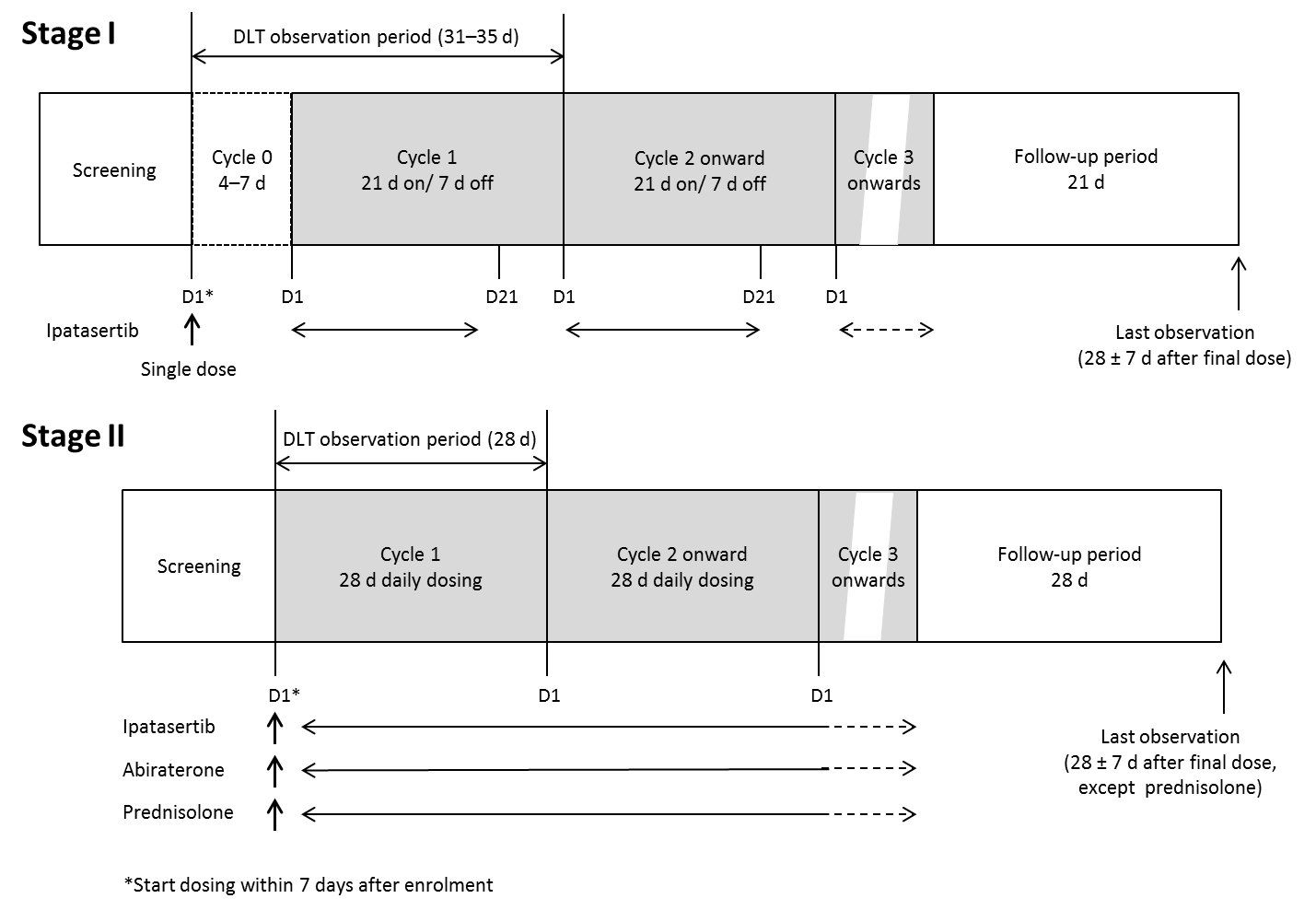

Supplement: Supplementary file 2 — Supplementary material 2 (DOCX 68 kb) [file 280_2019_3882_MOESM2_ESM.docx]
