# Supplementary material for: Phase I study of ipatasertib as a single agent and in combination with abiraterone plus prednisolone in Japanese patients with advanced solid tumors
Source: Cancer Chemother Pharmacol. 2019 Jun 21;84(2):393–404. doi: 10.1007/s00280-019-03882-7 (PMC6647215; doi:10.1007/s00280-019-03882-7)
Supplement: Supplementary file 4 — Supplementary material 4 (DOCX 15 kb) [file 280_2019_3882_MOESM4_ESM.docx]

**Online Resource 4.** Dose-limiting toxicities during Stage I and II

| Grade 4 neutropenia continuing for 5 days or longer |
| --- |
| Febrile neutropenia |
| Grade 4 thrombocytopenia, or grade 3 thrombocytopenia requiring platelet transfusion |
| Grade ≥4 anemia |
| Grade ≥3 non-hematologic toxicity (excluding transient electrolyte abnormalities). Abnormal liver function levels, hyperglycemia, hypercholesterolemia, hypertriglyceridemia, diarrhea, nausea, vomiting, and skin toxicity will be handled as DLTs only if they meet the following criteria.  Total bilirubin, AST, and ALT: grade ≥3 increase continuing for ≥3 days will be deemed a DLT. However, if the patient has a liver metastasis and the laboratory reference range was exceeded at enrollment, increase in total bilirubin to ≥5 times the upper limit of normal (ULN) or increase in AST or ALT to ≥7.5 times ULN will be deemed a DLT.  ALP: grade ≥3 increase continuing for 3 days or longer will be deemed a DLT. However, if the patient has a bone or liver metastasis and ULN was exceeded at enrollment, an increase to ≥10 times ULN will be deemed a DLT.  Fasting hyperglycemia: grade 4 event, or grade 3 event continuing for ≥1 week after start of treatment with an oral antidiabetic agent.  Hypercholesterolemia and hypertriglyceridemia: grade 4 (fasting) event continuing for ≥2 weeks after onset despite the use of an antihyperlipidemic agent.  Diarrhea, nausea, vomiting, skin toxicity: grade ≥3 event continuing for ≥1 week despite appropriate intervention. |
| Adverse events in Cycle 1 for which a causal relationship with ipatasertib cannot be ruled out that required suspension for >25% of the stipulated number of ipatasertib dosing days (a total of ≥6 days in Stage 1 and a total of ≥8 days in Stage 2) (the single-dose administration in Stage 1 is not included in the stipulated number of dosing days). |

ALP, alkaline phosphatase; ALT, alanine aminotransferase; AST, aspartate aminotransferase; DLT, dose-limiting toxicity; ULN, upper limit of normal
