# Supplementary material for: Phase I study of ipatasertib as a single agent and in combination with abiraterone plus prednisolone in Japanese patients with advanced solid tumors
Source: Cancer Chemother Pharmacol. 2019 Jun 21;84(2):393–404. doi: 10.1007/s00280-019-03882-7 (PMC6647215; doi:10.1007/s00280-019-03882-7)
Supplement: Supplementary file 5 — Supplementary material 5 (DOCX 15 kb) [file 280_2019_3882_MOESM5_ESM.docx]

**Online Resource 5.** Schedule for blood sampling for pharmacokinetic assessments.

| **Visit** | **Sampling time**  **(time window)** |
| --- | --- |
|  |  |
| Stage I | |
| Cycle 0 Day 1 | 5 min before dosing (−30 min) |
|  | 30 min after dosing (±5 min) |
|  | 1 hr after dosing (±5 min) |
|  | 2 hr after dosing (±5 min) |
|  | 3 hr after dosing (±10 min) |
|  | 4 hr after dosing (±10 min) |
|  | 6 hr after dosing (±30 min) |
| Cycle 0 Day 2 | 24 hr after dosing on Cycle 0 Day 1 (±2 hr) |
| Cycle 0 Day 3 | 48 hr after dosing on Cycle 0 Day 1 (±2 hr) |
| Cycle 0 Day 4 | 72 hr after dosing on Cycle 0 Day 1 (±2 hr) |
| Cycle 1 Day 1  (Cycle 0 Days 4–8) | 5 min before dosing (−30 min) |
|  | 2 hr after dosing (±5 min) |
|  | 6 hr after dosing (±1 hr) |
| Cycle 1 Day 8 | 5 min before dosing (−30 min) |
|  | 30 min after dosing (±5 min) |
|  | 1 hr after dosing (±5 min) |
|  | 2 hr after dosing (±5 min) |
|  | 3 hr after dosing (±10 min) |
|  | 4 hr after dosing (±10 min) |
|  | 6 hr after dosing (±30 min) |
| Cycle 1 Day 9 | 24 hr after dosing (±2 hr before dosing on Day 9) |
| Cycle 1 Day 15 | 5 min before dosing (−30 min) |
| Cycle 1 Day 22 | 24 hr after dosing on Cycle 1 Day 21 (±2 hr) |
| Cycle 2 Day 1 | 5 min before dosing (−30 min) |
| Cycle 2 Day 15 | 5 min before dosing (−30 min) |
| Cycle 4 Day 15 | 5 min before dosing (−30 min) |
| Cycle 6 Day 15 | 5 min before dosing (−30 min) |
| Stage II | |
| Cycle 1 Day 1 | 5 min before dosing (−30 min) |
|  | 1 hr after dosing (±5 min) |
|  | 2 hr after dosing (±5 min) |
|  | 4 hr after dosing (±10 min) |
|  | 6 hr after dosing (±30 min) |
| Cycle 1 Day 2 | 24 hr after dosing (±2 hr before dosing on Day 2) |
| Cycle 1 Day 15 | 5 min before dosing (−30 min) |
|  | 1 hr after dosing (±5 min) |
|  | 2 hr after dosing (±5 min) |
|  | 4 hr after dosing (±10 min) |
|  | 6 hr after dosing (±30 min) |
| Cycle 1 Day 16 | 24 hr after dosing (±2 hr before dosing on Day 16) |
| Cycle 2 Day 1 | 5 min before dosing (−30 min) |
| Cycle 3 Day 1 | 5 min before dosing (−30 min) |
| Cycle 6 Day 1 | 5 min before dosing (−30 min) |
